# Trends of hospitalizations, fatality rate and costs for acute myocardial infarction among Spanish diabetic adults, 2001-2006

**DOI:** 10.1186/1472-6963-10-59

**Published:** 2010-03-08

**Authors:** Ana Lopez-de-Andres, Valentín Hernández-Barrera, Pilar Carrasco-Garrido, Jesús Esteban-Hernandez, Ángel Gil-de-Miguel, Rodrigo Jiménez-García

**Affiliations:** 1 Preventive Medicine and Public Health Teaching and Research Unit, Health Sciences Faculty, Rey Juan Carlos University, Avda de Atenas s/n, Alcorcón 28922 Madrid, Spain

## Abstract

**Background:**

Acute myocardial infarction (AMI) is one of the more frequent reasons diabetic patients are admitted to hospital, and there are reports that the long-term prognosis after an AMI is much worse in these patients than in non-diabetic patients. This study aims to compare hospital admissions and costs in Spanish diabetic and non-diabetic subjects due to AMI during the period 2001-2006.

**Methods:**

We conducted a retrospective study of 6 years of national hospitalization data associated with diabetes using the Minimum Basic Data Set. National hospitalization rates were calculated for AMI among diabetic and non-diabetic adults. Fatality rates, mean hospital stay and direct medical costs related to hospitalization were analyzed. Costs were calculated using Diagnosis-Related Groups for AMI in diabetics and non-diabetics patients.

**Results:**

During the study period, a total of 307,099 patients with AMI were admitted to Spanish hospitals. Diabetic patients made up 29.6% of the total. The estimated incidence due to AMI in diabetics increased from 54.7 cases per 100,000 in 2001 to 64.1 in 2006. Diabetic patients had significantly higher mortality than nondiabetic patients after adjusting for age, gender, and year (OR 1.11 [95% CI, 1.08-1.14]). The cost among diabetic patients increased by 21.3% from 2001 to 2006.

**Conclusions:**

Diabetic patients have higher rates of hospital admission and fatality rates during the hospitalization after an AMI than nondiabetic patients. Diabetic adults who have suffered an AMI have a greater than expected increase in direct hospital costs over the period 2001-2006.

## Background

The prevalence of diabetes in Spain has increased during the last 10 years. A recent review shows that it varies from 10% to 15% [1] according to the study, population, and diagnostic method. Other group shows that the prevalence of diabetes in Spain in individuals aged < 65 years is approximately 6%, a percentage that increases with age [2].

In Spain, the Diabetes Strategy of the National Health System (Ministry of Health and Consumer Affairs) calculates the mortality rate of diabetes to range from 12.75 to 30.37 deaths per 100,000 inhabitants per year. Coronary disease is the main cause of death among diabetes patients, and 75% of patients with diabetes die mainly of coronary disease [3].

Cardiovascular disease and diabetes are clearly related. Indeed, several cohort studies show that diabetic patients are at greater risk of experiencing a coronary event than the general population [4,5]. Therefore, as a complication of diabetes, cardiovascular disease is associated with a greater proportion of direct costs [6]. Gandra et al (2006) showed that health care costs triple in diabetic patients with cardiovascular complications [7].

Acute myocardial infarction (AMI) is one of the reasons diabetic patients with cardiovascular complications are admitted to hospital, and there are reports that the long-term prognosis after an AMI is much worse in diabetic patients than in nondiabetic patients [8,9]. In fact, the mortality rate for AMI is approximately double in diabetic patients [9].

However, despite the association between diabetes and AMI, few studies quantitatively assess the role of diabetes in the morbidity, mortality, and hospital costs of coronary disease. In the present study, we aim to determine how hospital costs and mortality in Spain evolve in diabetic patients admitted with AMI between 2001 and 2006.

## Methods

A retrospective, descriptive, epidemiologic study was conducted, using the Minimum Basic Data Set (MBDS) as the data source. The MBDS is a national hospital admission database containing discharge data abstracted for at least 95% of all Spanish acute care hospitalizations. It is managed by the Spanish Ministry of Health and Consumer Affairs [10] and includes the following data: hospital details; patient's name, date of birth, gender, place of residence, and date of admission; surgical and obstetric procedures; other procedures; and date and type of discharge.

Diagnostic coding was based on the International Classification of Diseases (ICD-9-CM). Each contact is recorded with one o more diagnosis code in ICD-9-CM. Myocardial infarction was defined by the main medical diagnosis with codes 410.0-410.9. Patients were then further defined by presence/absence of diabetes (ICD-9-CM code 250) using secondary medical diagnosis (in any of its 13 sections).

Incidence of hospitalization due to AMI among subjects with or without diabetes was calculated using the Spanish National Statistics Institute census projections from the year 2001 [11].

Mean hospital stay due to AMI in patients with and without diabetes was calculated overall and by age group. We also analyzed mortality during hospitalization among patients with and without diabetes.

The direct costs to the National Health System were calculated using Diagnosis-Related Groups (DRG) for this disease. According to the DRG reimbursement system, every hospitalized patient belongs to a group of diagnostically homogeneous cases; therefore, patients within each category are similar in clinical terms, and are expected to use the same level of hospital resources. As a result, patients in the same DRG are assigned the same reimbursement charges [10].

We use a specific DRG for AMI (DRG 121: CIRCULATORY DISORDERS W AMI & MAJOR COMP, DISCHARGED ALIVE) and for diabetes (DRG 294: DIABETES AGE >35) (All patients -DRG version 22.0) [10].

In order to calculate the cost per patient above the Retail Price Index (RPI), we used the RPI section of the Spanish National Statistics Institute web page. The RPI measures the development of prices of goods and services consumed in Spain [12].

The cost per patient without diabetes was aged and sex adjusted using the diabetic population as a reference. Quantitative variables were expressed as the mean ± standard deviation and qualitative variables were expressed as frequencies. The chi-square test was used to compare categorical variables, and t tests and an ANOVA to compare means. Statistical significance was set at P < 0.05 (two-tailed). To compare the incidence of AMI for patients with and without diabetes, and the time course for both groups, we used multivariate Poisson regression analysis. We used logistic regression models to analyze mortality. The logistic regression multivariate model was built using the "enter modelling" method." The dependent variable was death (yes/no) and the independent variables included: year, sex and age.

All statistical analyses were performed using the Stata statistics computer software package.

## Results

A total of 307,099 patients with AMI were admitted to Spanish hospitals covered by the MBDS during the 6-year study period. The mean age was 68.5 ± 13.1 years (median 71 years), and 69% were men. Diabetic patients made up 29.6% of the total (90,835).

Table 1 shows the annual hospital admission rates according to gender, for both diabetics and nondiabetics subjects. These rates increased until 2003, when they began to fall: the mean stay fell from 10.4 days in 2001 to 9.6 days in 2006 for diabetic patients (p ≤ 0.05), and from 9.8 days in 2001 to 8.4 days in 2006 for nondiabetic patients (p ≤ 0.05). During admission, 12.4% of diabetics adults and 9.9% of nondiabetics died (p ≤ 0.05).

**Table 1:** Hospital admissions by acute myocardial infarction among subjects with or without diabetes for years 2001-2006.

		With Diabetes				Without Diabetes			
**Year**	**Sex**	**Total**	Inc.*	**M.S. (SD)†**	**%F.R. †**	**Total**	Inc.*	**M.S. (SD)†**	**%F.R. †**

2001	Men	7,030	66.5	10.3(8.0)	10.7	25,127	237.7	9.6(10.1)	9.2
	
	Female	5,206	44.1	10.5(9.1)	16.7	9,016	76.3	9.8(8.99	16.7
	
	Both	12,236	54.7	10.4(8.4)	13.2	34,143	152.5	9.8(9.4)	11.2

2002	Men	8,066	75.9	10.5(9.1)	11.1	26,945	253.5	9.7(9.6)	8.6
	
	Female	5,802	48.9	10.6(9.0)	17.5	9,970	84.1	9.9(9.2)	15.7
	
	Both	13,868	61.7	10.5(9.1)	13.8	36,915	164.2	9.7(9.5)	10.5

2003	Men	9,332	85.6	10.3(8.7)	10.2	26,927	247.1	9.2(8.6)	8.5
	
	Female	6,636	54.7	10.4(9.5)	16.6	9,966	82.2	9.3(8.3)	15.4
	
	Both	15,968	69.4	10.3(9.1)	12.9	36,893	160.3	9.2(8.5)	10.3

2004	Men	9,685	86.7	9.8(8.1)	9.6	26,833	240.2	9.0(9.8)	7.7
	
	Female	6,716	54.2	10.2(8.4)	15.0	9,726	78.5	9.3(11.5)	15.2
	
	Both	16,401	69.6	9.9(8.2)	11.8	36,559	155.2	9.0 (10.3)	9.7

2005	Men	10,026	87.7	9.6(8.5)	10.2	26,369	230.6	8.6(8.8)	7.5
	
	Female	6,582	52.0)	9.9(8.2)	15.1	9,819	77.6	9.2(8.5)	13.8
	
	Both	16,608	69.0	9.7(8.4)	12.1	36,188	150.3	8.8(8.7)	9.2

2006	Men	9,659	82.5	9.4(8.8)	9.0	26,038	222.6)	8.3(8.3)	6.8
	
	Female	6,095	47.2	9.9(8.3)	14.5	9,528	73.8	8.7(8.7)	13.2
	
	Both	15,754	64.1	9.6(8.6)	11.2	35,566	144.6	8.4(8.4)	8.5

Total	Men	53,798	81.0	10.0(8.6)	10.1	158,239	238.3	9.2(9.1)	8.0
	
	Female	37,037	50.2	10.3(8.7)	15.9	58,025	78.6	9.5(9.3)	15.0
	
	Both	90,835	64.8	10.1(8.7)	12.4	216,264	154.4	9.2(9.2)	9.9

*Inc: Incidence per 100,000. Incidence was calculated using the Spanish National Statistics Institute census projections from the year 2001.

†M.S. (SD): Mean Stay (Standard Deviation). %F.R.: Fatality rate.

The incidence of hospital admissions due to AMI in diabetic subjects increased from 54.7 cases per 100,000 inhabitants in 2001 to 64.1 cases per 100,000 inhabitants in 2006. Mean stay decreased significantly (10.3 ± 8.0 days for diabetic men and 10.5 ± 9.1 days for diabetic women in 2001 compared with 9.4 ± 8.8 days for diabetic men and 9.9 ± 8.3 days for diabetic women in 2006), as did fatality rate (10.7% in diabetic men and 16.7% in diabetic women in 2001 compared with 9.0% in diabetic men and 14.5% in diabetic women in 2006).

By age group, incidence increased with age in diabetic patients (17.7 per 100,000 inhabitants for aged 35-60 years vs. 205.4 per 100,000 inhabitants for aged over 81 years) and was greater for men in every year. During each year of the study, the mean stay was greater in diabetic women than men. Fatality rate was greater in diabetic women than men in all age groups.

Table 2 presents the results of the multivariate analysis. After controlling for possible confounders, we observed that the incidence of admissions among diabetic patients increased significantly during the 6-year study period (IRR 1.16 95% CI 1.14-1.19). Diabetic patients had significantly higher mortality than nondiabetic patients after adjusting for age, gender, and year (OR = 1.11; 95% CI, 1.08-1.14), and mortality among diabetic patients increased significantly with age and fell during the study period. Mortality was significantly greater in diabetic women than in men (OR = 1.22;95% CI, 1.17-1.28).

**Table 2:** Multivariate analysis of the factors associated with incidence and mortality after acute myocardial infarction.

		Incidence (IRR)*		**Fatality (OR)**^**†**^	
		
		Diabetic patients	Non diabetic patients	Diabetic patients	Non diabetic patients
**Age (years)**	**35-60**	1	1	1	1
	
	**61-70**	5.40 (5.29-5.51)	2.59 (2.56-2.62)	2.18 (1.98-2.40)	2.36 (2.22-2.51)
	
	**71-80**	10.54 (10.35-10.79)	4.34 (4.29-4.38)	3.91 (3.57-4.27)	5.00 (4.74-5.27)
	
	**≥81**	12.87 (12.59-13.15)	7.02 (6.94-7.11)	6.55 (5.98-7.18)	10.31 (9.77-10.82)

**Sex**	**Men**	1	1	1	1
	
	**Female**	0.52 (0.51-0.52)	0.28 (0.28-0.28)	1.22 (1.17-1.28)	1.18 (1.14-1.22)

**Year**	**2001**	1	1	1	1
	
	**2002**	1.12 (1.10-1.15)	1.07 (1.05-1.09)	1.03 (0.96-1.11)	0.90 (0.85-0.94)
	
	**2003**	1.26 (1.24-1.29)	1.04 (1.03-1.06)	0.93 (0.86-1.00)	0.88 (0.84-0.92)
	
	**2004**	1.27 (1.24-1.30)	1.01 (0.99-1.02)	0.84 (0.78-0.90)	0.81 (0.77-0.86)
	
	**2005**	1.26 (1.23-1.29)	0.97 (0.96-0.99)	0.86 (0.80-0.92)	0.76 (0.72-0.80)
	
	**2006**	1.16 (1.14-1.19)	0.93 (0.92-0.95)	0.78 (0.73-0.84)	0.70 (0.66-0.74)

* Calculated using multivariate Poisson regression: Incidence Rate Ratios (IRR). ^† ^Calculate using logistic regression models:Odds Ratio (OR). The logistic regression multivariate model and Poisson regression model were built using as dependent variables "death (yes/no)" and "Incidence of AMI" respectively, and as independent variables year, sex and age.

Table 3 presents how the hospitalization costs for AMI evolved during the study period in diabetic and non-diabetic patients. Using the DRG for the study diseases, the estimated cost per diabetic patient and year was €4,440 in 2001 and €6,228 in 2006 (total cost was €54,334,000 in 2001 and €98,075,000 in 2006). The cost of admissions for diabetic patients increased significantly (p ≤ 0.05) along the study period. When the RPI effect was eliminated, the cost in diabetic patients increased by 19.5% from 2001 to 2006.

**Table 3:** Hospitalization costs among subjects who have suffered a myocardial infarction according to age groups and year.

		Year					
**Diabetic patients**	Age, years*	**2001**	**2002**	**2003**	**2004**	**2005**	**2006**
	
	**35-60**	9,917	12,134	14,941	16,470	17,089	19,677
	
	**61-70**	15,575	18,365	21,509	22,174	22,966	23,597
	
	**71-80**	20,250	25,900	32,086	34,171	35,042	37,215
	
	**>81**	8,590	11,213	14,253	16,458	17,525	17,584
	
	**Total**	54,334	67,614	82,792	89,274	92,623	98,075
	
	**P/A**	4,440	4,875	5,187	5,443	5,577	6,228

	**Difference above RPI**	-----	297	132	111	-34	417

**Non diabetic patients**	Age, years*						
	
	**35-60**	51,544	59,523	66,500	69,033	69,089	81,154
	
	**61-70**	39,859	42,942	45,521	44,433	44,733	49,150
	
	**71-80**	45,581	54,196	55,246	59,529	59,163	60,056
	
	**>81**	22,583	29,075	30,669	33,342	35,405	37,109
	
	**Total**	159,568	185,736	197,937	206,338	208,392	227,470
	
	**P/A**	4,625	5,005	5,288	5,587	5,718	6,283
	
	**Difference above RPI**	-----	211	150	125	-63	393

* Thousands of Euros. P/A: Total cost per patient and year (Euros). RPI: Retail Price Index

The cost per patient without diabetes has also increased across the study period from €4,625 in 2001 to €6,283 in 2006. The cost was higher among those without versus those with diabetes in all the years of the study. When the RPI effect was eliminated, the cost in non-diabetic patients increased by 28,2% from 2001 to 2006.

## Discussion

### Main Findings

Our results show that approximately 30% of Spanish adults who experience an AMI have an associated diagnosis of diabetes. The results of the 1998 PREVESE study revealed that 22.6% of patients admitted to hospital for AMI were diabetics [13]. This discrepancy with our results could be explained by the fact that the prevalence of diabetes has increased in Spain during the last 10 years [1].

These results are also consistent with those of Fang et al (2006), who showed that the hospital workload generated by AMI is greater in diabetic patients than in nondiabetic patients [14].

During the study period, overall mortality as a consequence of AMI decreased in both diabetic and nondiabetic patients. Booth et al (2006) showed that both diabetics and nondiabetics who have experienced an AMI have lower mortality rates over time, suggesting that the care and management of AMI patients have improved in recent years [15].

After adjusting for age and gender, we observed that the in-hospital mortality for diabetic patients who have experienced an AMI is significantly greater (11%) than for nondiabetic patients. Several studies report that diabetics who experience an AMI have higher fatality rates than nondiabetics [4,5,16]. This could be due to the fact that these patients have a worse clinical status than nondiabetics or are at a greater risk of complications [15].

The outcome of diabetic patients over time, both in terms of admissions and in terms of mortality after AMI, was less favorable in those aged ≥65 years than in younger patients. Svensson et al (2007) showed that mortality at 7 years post-AMI was 59.2% in diabetic patients aged over 65 years and 46.3% in those aged 55-64 years [9]. On the other hand, another study showed that in-hospital mortality due to AMI in diabetics aged over 65 years fell at a lower rate than in diabetic patients aged less than 65 years [15].

The literature clearly shows that women have a lower incidence of AMI than men [17,18]. However, hospitalized women have been reported to undergo fewer diagnostic or revascularization procedures, and therefore have a higher mortality rate [19]. In our study, diabetic women had significantly higher mortality rates than diabetic men. These results are consistent with those of other studies that specifically analyze the interaction between gender and diabetes [9,18,19]. In the Secondary Prevention Reinfarction Israeli Nifedipine Trial (SPRINT) study, diabetes was a significant predictor of hospital mortality after AMI in women, but not in men. Also, in the US Second National Registry of Myocardial Infarction, women were at greater risk of hospital death than men, even after adjusting for comorbid conditions and treatment differences. These data suggest that hemodynamic and other mechanisms associated with the onset of acute infarction may be compromised to a greater extent in women than in men with diabetes [20,21].

Our study revealed an inverse relationship between mean hospital stay and costs over time in patients who experienced an AMI. This could be due to several factors. First, recent advances in therapy have improved reperfusion treatment and led to more widespread use of antithrombotic drugs and diagnosis methods thus reducing hospital stay while increasing costs [22]. Second, 40% of deaths by AMI in Spain occur in patients who do not reach hospital, but fortunately this percentage is decreasing over time and therefore more severe cases now reach the hospital and increase the cost [23]. Third, older age and prevalence of comorbid conditions could also explain the increased costs of hospital stay after an AMI [24].

Direct medical costs per patient did not differ significantly between those with and without diabetes. Other authors find that diabetes increases healthcare costs among patients hospitalized with cardiovascular disease [25].

In our population costs were mainly generated by elderly patients (> 70 years), thus confirming the results of previous studies, which show that patients aged over 65 years account for approximately two-thirds of all diabetes-associated medical costs [8].

### Study Limitations

The strength of our study lies in its large sample size and standardized methodology. Nevertheless, it does present a series of limitations. First, our database was a National Health Service data set of information on hospital discharges; therefore, it contains scant clinical information (eg, pharmacologic treatment is not recorded). The data in the MBDS are anonymous, with the result that it is impossible to determine whether the same patient was admitted more than once during the same year. However, this data set, which was introduced in Spain in 1982, is a mandatory register, and its coverage is estimated to be more than 95% [10]. Furthermore, it provides useful information on hospital management of AMI, since it brings together data from all admissions due to AMI, regardless of the characteristics of the attending hospital or the department to which the patient is admitted.

Third, although the financial burden of diabetes in patients admitted to hospital with AMI is considerable, we feel that the real cost of diabetes is underestimated, because many patients are not diagnosed as having diabetes, or diabetes is not included in the diagnosis on admission [25].

A complete classification of cases in type 1 and type 2 diabetes is not possible based on the information available in registers; hence the type of diabetes is not considered in this report.

Costs were calculated using the DRG for the disease. Although DRG have been widely used as a patient classification system for hospital cost analysis, they present a series of limitations [26], including significant internal variability in comorbid conditions and a lack of sensitivity for different medical practices used in similar diseases. In addition, the indication for diagnosis and therapeutic procedures has not always been evaluated [26].

## Conclusions

Our study provides updated information on the impact of AMI in diabetic patients and nondiabetic patients, and on the costs associated with admissions to hospital due to this condition over a 6-year period in Spain.

In Spain the continuing increase in the prevalence of diabetes and the lack of improvement in the control of cardiovascular risk factors and health-related behaviors call for greater dedication on the part of persons with diabetes and the health care community [1-3,13,27]. More effective interventions to improve this situation are clearly needed and could include: empowering patients with information to improve the quality of care they receive, their lifestyles and health outcomes; and ongoing monitoring and measurement of cardiovascular risk factors and unhealthy behaviors [1-3,13,27]. Only improving the education, prevention and quality of care we could reduce the incidence, mortality and burden of AMI among Spanish diabetes sufferers [1-3,13,27].

We conclude that diabetic patients have higher rates of hospital admission and fatality rates during the hospitalization after an AMI than nondiabetic patients, Diabetic adults who have suffered an AMI have a greater than expected increase in direct hospital costs over the period 2001-2006.

## Competing interests

The authors declare that they have no competing interests.

## Authors' contributions

ALA has participated in conceive the study, has made substantial contribution to analysis and interpretation of the data, and has been involving in writing the manuscript.

VHB has made substantial contribution to analysis the data. PCG has been involving in writing the manuscript and revising it critically for important intellectual content. JEH has made substantial contribution to analysis the data. AGM has been involving in writing the manuscript and revising it critically for important intellectual content. RJG has conceived the study, has been involving in writing and participated in its design and coordination and helped to draft the manuscript and revising it critically for important intellectual content. All authors read and approved the final manuscript.

## Pre-publication history

The pre-publication history for this paper can be accessed here:

http://www.biomedcentral.com/1472-6963/10/59/prepub
